# Improving the Accuracy of Flow Cytometric Quantification of Microbial Populations in Sediments: Importance of Cell Staining Procedures

**DOI:** 10.3389/fmicb.2019.00720

**Published:** 2019-04-09

**Authors:** Longhui Deng, Annika Fiskal, Xingguo Han, Nathalie Dubois, Stefano Michele Bernasconi, Mark Alexander Lever

**Affiliations:** ^1^Institute of Biogeochemistry and Pollutant Dynamics, ETH Zürich, Zurich, Switzerland; ^2^Surface Waters Research-Management, Eawag, Swiss Federal Institute of Aquatic Science and Technology, Dübendorf, Switzerland; ^3^Department of Earth Sciences, ETH Zürich, Zurich, Switzerland

**Keywords:** microbial populations, lacustrine, marine, cell counts, staining technique, flow cytometry, epifluorescence microscopy

## Abstract

The accuracy of flow cytometric (FCM) quantifications of microbial populations in sediments varies with FCM settings, cell extraction and staining protocols, as well as sample types. In the present study, we improve the accuracy of FCM for enumerating microorganisms inhabiting diverse lake and marine sediment types based on extensive tests with FCM settings, extraction buffer chemical compositions, cell separation methods, and staining procedures. Tests on the FCM settings, (e.g., acquisition time, rates of events) and salinity of extraction solutions show minor impacts on FCM enumerations and yields of cell extraction, respectively. Existing methods involving hydrofluoric acid (HF) treatment to release sediment-attached cells into solution prove effective on both marine and freshwater samples. Yet, different staining techniques (direct staining of cell extracts, staining of membrane-filtered cell extracts) produce clear differences in cell number estimates. We demonstrate that, while labor-intensive membrane-staining generates high cell staining efficiency and accurate cell counts that are consistent across FCM and epifluorescence microscopy-based (EFM) quantification methods, accurate cell counts determined by more time- and labor-efficient direct staining require consideration of dye concentration, sample dilution, and lithology. Yet, good agreement between the two staining methods can be achieved through sample-specific adjustments of dye concentrations and sample dilutions during direct staining. We thus present a complete protocol for FCM-based cell quantification, that includes all steps from the initial sample fixation to the final enumeration, with recommendations for buffer compositions, direct and membrane-based staining procedures, and the final FCM assay. This protocol is versatile, accurate, and reliable, as is evident from good agreement with cell quantifications by EFM and quantitative polymerase chain reaction (qPCR) of 16S rRNA genes across a wide range of sedimentary sample types.

## Introduction

Microorganisms are ubiquitous in marine and freshwater sediments and play important roles in global elemental cycles, including the carbon and nitrogen cycles. Previous estimates of microbial abundance in subseafloor sediment based on different techniques vary from 2.9–50 × 10^29^ cells, of which the lower boundary is comparable to global cell numbers in seawater and soil, whereas the upper boundary approaches the total microbial abundance elsewhere on Earth (Whitman et al., 1998; Lipp et al., 2008; Kallmeyer et al., 2012; Parkes et al., 2014). Although lake sediments cover a much smaller percentage of Earth’s surface area than marine sediments, the annual organic carbon burial in lake sediments is comparable to that in marine sediments (Dean and Gorham, 1998; Cole et al., 2007; Mendonça et al., 2017), and microorganisms are abundant in freshwater sediment (Leahy and Colwell, 1990; Haglund et al., 2003). Thus, lake sediments may also host a significant fraction of global microbial biomass.

A reliable and fast quantification method is critical for estimating microbial population size in both marine and freshwater sediment. Numerous techniques have been used previously, e.g., direct epifluorescence microscopic counting of cells (Kepner and Pratt, 1994; Kallmeyer et al., 2008), Fluorescence *in situ* hybridization (FISH, Llobet-Brossa et al., 1998; Bouvier and Del Giorgio, 2003), catalyzed reporter deposition-FISH (CARD-FISH, Pernthaler et al., 2002; Schippers et al., 2005), quantitative PCR (qPCR, Schippers and Neretin, 2006; Chen et al., 2017), adenosine tri-phosphate (ATP) measurement (Frossard et al., 2016), and lipid quantification (White et al., 1979; Lipp et al., 2008). Yet, the results derived from different techniques often show limited agreement, even when the same samples are studied (Lloyd et al., 2013; Buongiorno et al., 2017).

Direct counts of fluorescence-stained microbial cells by epifluorescence microscopy-based (EFM) have been used to quantify microbial population size in natural samples since the early 1970s (Babiuk and Paul, 1970). Various fluorescent dyes, such as acridine orange (AO; Francisco et al., 1973), 4′,6-diamidino-2-phenylindole (DAPI; Porter and Feig, 1980), SYBR Green I (SYBR-I; Noble and Fuhrman, 1998), and SYBR Green II (SYBR-II; Weinbauer et al., 1998) have been applied to stain intracellular nucleic acids, and thereby distinguish microbial cells from background. Among these dyes, SYBR-I is frequently used on natural samples, because of its high binding affinity to both DNA and RNA, which leads to bright fluorescence (Karlsen et al., 1995; Marie et al., 1997). One challenge of EFM enumeration in sediments has been the discrimination of stained microbial cells from unspecifically stained viral particles, detritus, e.g., containing extracellular DNA, or microorganism-sized minerals (Noble and Fuhrman, 1998; Soler et al., 2008). Auto-fluorescence of photosynthetic pigments, e.g., phycobilin and chlorophyll-a, diatom frustules, or mineral particles can also contribute to false positive signals (Marie et al., 1997). To reduce these matrix effects, protocols for cell detachment from sedimentary particles, e.g., involving chemical (Lunau et al., 2005; Duhamel and Jacquet, 2006), mechanical (Ellery and Schleyer, 1984; Epstein and Rossel, 1995; Buesing and Gessner, 2002), or enzymatic treatment (Böckelmann et al., 2003; Kallmeyer et al., 2008) have been applied and frequently combined with direct centrifugation (Lunau et al., 2005; Lavergne et al., 2014), density-gradient centrifugation (Kallmeyer et al., 2008; Morono et al., 2013), and/or filtration (Duhamel and Jacquet, 2006). Dissolution and disintegration of silicate clay, silt, or sand using hydrofluoric acid (HF) has turned out to be particularly effective in reducing interfering signals from sediment particles and extracting cells that were initially firmly attached to these mineral matrices (Boenigk, 2004; Morono et al., 2009; Langerhuus et al., 2012).

To date direct counting of microbial cells by EFM has been successfully applied to a wide range of natural samples, including soils (Dobbins et al., 1992; Richaume et al., 1993), marine sediments (Parkes et al., 1994; Schippers et al., 2005; Inagaki et al., 2006; Kallmeyer et al., 2012), freshwater sediments (Haglund et al., 2003; Amalfitano and Fazi, 2008), and aquifers (Wilson et al., 1983; Balkwill et al., 1988). As a standard approach to quantify microbial populations in sediment, however, EFM-based enumeration has its own limitations: it is time- and labor-intensive, it includes human biases, and the detection of small cells (<0.5 μm) and cells that are hidden under particles can be challenging. Although a high-throughput enumeration technique based on a robotic slide-shifter system, combined with automatic photography and image analyses, has been developed, this customized setup is difficult and costly to reproduce in other laboratories (Morono et al., 2009).

Since the 1980s, techniques involving fluorescent staining combined with FCM have been developed and widely used for enumerating microbial cells in natural water samples (Porter et al., 1993; Lebaron and Joux, 1994; Gasol and Del Giorgio, 2000; Hammes and Egli, 2010; Wang et al., 2010; Irvine-Fynn et al., 2012). Despite the high-sensitivity and high-throughput features of FCM, few reports exist describing the application of FCM to sediments (Lake sediments: Duhamel and Jacquet, 2006; Marine sediments: Morono et al., 2013; Lavergne et al., 2014; Streambed sediments: Amalfitano and Fazi, 2008; Amalfitano et al., 2009; or multiple types of samples: Frossard et al., 2016). The accuracy of FCM enumeration is often unclear, as ratios of FCM- vs. EFM-based enumerations of microbial cells can vary significantly among different types of samples, (e.g., 0.3 to 7.5 in Frossard et al., 2016). These discrepancies have been attributed to observer bias, inclusion of fluorescence-stained background particles, decreases in fluorescence during EFM counting, or higher cell detection sensitivity of FCM, (e.g., Lavergne et al., 2014; Frossard et al., 2016).

One potentially overlooked factor that is influencing the accuracy of FCM enumeration is the staining efficiency of microbial cells. Direct staining, i.e., staining the cells by directly adding fluorescent dye to cell extracts is a widely used technique for staining microbial pure-cultures, bacterioplankton, as well as sedimentary microorganisms, (e.g., Berney et al., 2007; Gasol and Del Giorgio, 2000; Lavergne et al., 2014). Yet, quantifications of sedimentary cells after direct staining can be challenging because non-cell particles may “compete” with microbial cells for dye molecules by unspecific absorption and then be mistakenly counted as cells (Morono et al., 2013). Another method, membrane staining, whereby cells are stained after filtration of cells onto a membrane filter, represents a routine procedure during EFM enumeration, (e.g., Weinbauer et al., 1998). One study (Morono et al., 2013) has shown this method to be compatible with FCM, if membrane-stained cells are washed off the membrane prior to FCM counting. Yet, membrane-staining followed by membrane washing is considerably more costly and labor-intensive than direct staining, and also more prone to contamination due to the various filtration steps and additional solutions used during these. Thus, a direct staining protocol that allows for reliable discriminations between cells and background particles and for accurate quantification of microbial populations in sediments would potentially be advantageous. Besides the staining procedure, there are several additional factors that might influence the accuracy of FCM enumeration, such as the FCM assay itself, and the chemical composition of cell extraction solutions. Whether and how much these factors individually and/or jointly affect the staining efficiency and accuracy of FCM enumerations still needs to be addressed.

Here we examine the accuracy of FCM enumeration of microbial populations in lake and marine sediments by performing tests involving different FCM settings (flow speed, acquisition time, rates of events), salinities of extraction solutions (NaCl concentration), cell separation methods (HF-based, density-gradient centrifugation), and staining methods (direct staining versus membrane staining). While the FCM settings and solution salinities tested only have a small effect on cell counts, FCM counts based on time-efficient direct staining do not consistently agree with those based on membrane staining. We show that suboptimal dye concentrations and sediment dilutions during direct staining are the reason for these discrepancies. Fortunately, this issue can be resolved through minor, sample type-specific optimizations during direct staining. We demonstrate the efficacy of the optimized direct staining method based on diverse surface and subsurface sediment sample types from freshwater lakes (oligotrophic to highly eutrophic) and marine environments (intertidal, continental shelf, cold seep, hydrothermal), where FCM-based cell counts with direct staining not only show good agreement with ones after membrane staining, but are also reproducible by other cell quantification methods, including EFM and qPCR.

## Materials and Methods

### Sample Collection

An overview of all sediment samples and sediment characteristics tested can be found in Table 1. Sediment samples with different total organic carbon (TOC) contents and grain size distributions were collected for developing a widely applicable method of cell enumeration. Lake sediment cores were obtained in June–July 2016 from the oligotrophic Lake Lucerne, the mesotrophic Lake Zurich, and the eutrophic Lake Greifen, Lake Zug, and Lake Baldegg in central Switzerland using a UWITEC gravity corer. Marine sediment samples were collected at or near Guaymas Basin, northern Gulf of California, using a gravity corer or a video-guided multicorer during the *R/V Sonne* Expedition SO241 in June–July 2015. Intertidal samples from False Bay, San Juan Island, United States were collected using push cores. From all cores, only the uncontaminated core centers were sampled. In addition, *Escherichia coli* were grown in Luria-Bertani media (Sezonov et al., 2007) for ∼20 h at 37°C to a final concentration of ∼2.5 × 10^8^ cells mL^−1^.

**Table 1:** Overview of site locations and bulk sedimentary characteristics of samples.

Samples	Latitude (N)	Longitude	Sample depths (cm)	TOC (%)	Clay^2^ (0-4 μm)	Silt^2^ (4-63 μm)	Sand^2^ (63-1000 μm)	D/S ratio^3^
**Lake Sediment**								
Lake Lucerne (LL)	47° 00.05′	8° 20.22′ E	1–1.5, 1.5–2, 6–8, 28–32	2.8 ± 0.9	39.7	48.9	11.3	4,000
Lake Zurich (LZ)	47° 17.00′	8° 35.62′ E	1.5–2, 4–6, 28–32	2.8 ± 0.2	46.2	49.8	3.9	6,000
Lake Greifen (LG)	47° 21.13′	8° 40.51′ E	1–1.5, 6–8, 28–32	2.4 ± 0.7	35.8	62.5	1.8	8,000
Lake Zug (LZG)	47° 10.27′	8° 30.04′ E	1–1.5, 6–8, 20–24	2.5 ± 0.6	52.2	47.5	0.2	8,000
Lake Baldegg (LB)	47° 11.93′	8° 15.61′ E	1–1.5, 4–6, 28–32	2.5 ± 0.3	38.9	59.7	1.4	6,000
**Marine Sediment**								
False Bay (intertidal)	48° 29.20′	123° 04.48′ W	0–2, 6–8, 26–30	0.2 ± 0.1	2.1	42.0	56.0	2,000
Guaymas Basin (hydrothermal)	27° 24.58′	111° 23.27′ W	0–1, 6–8, 14–16	1.6 ± 0.7	39.6	51.4	9.0	8,000
Guaymas Basin (OMZ^1^)	27° 42.41′	111° 13.66′ W	0–5, 350, 900	2.8 ± 0.5	43.1	52.7	4.1	6,000
Guaymas Basin (cold seep)	27° 28.19′	111° 28.36′ W	33, 233, 483	4.4 ± 0.6	38.8	57.3	3.9	4,000
N Gulf of California (coastal)	27° 55.01′	111° 01.13′ W	0–1, 4–6, 16–18	0.5 ± 0.1	10.3	62.7	27.0	2,000

*^1^OMZ, oxygen minimum zone, ^2^all measurements were on lake samples from 1 to 2 cm sediment depth, marine samples from the topmost depth. ^3^D/S ratio is the ratio of dye concentration to the sediment volume used for staining, for more information see Supplementary Table S1.*

### Preparation of Positive and Negative Controls

Negative controls were produced by autoclaving lake sediments at 121°C for 2 × 40 min, after which cell counts were below detection (<10^5^ cells cm^−3^) in these sediments. *E. coli* cells were fixed as described above and added at a known concentration (∼2.5 × 10^8^ cells mL^−1^) to autoclave-sterilized lake and marine sediments to serve as positive controls. Positive and negative controls were prepared in triplicate, and fixed and stored as natural samples (see below).

### Procedures Tested or Fine-Tuned in This Study

#### Cell Fixation and Extraction

Previous studies suggest to adjust the salinity of extraction solutions according to the sample salinity to reduce the osmotic pressure on cells, (e.g., Kallmeyer et al., 2008). Yet, when working with samples with a range of natural salinities, it requires considerable effort to prepare extraction solutions of with salinities corresponding to all sample salinities. Thus, we tested the potential negative effect of 3% (*w/v*) NaCl in cell fixation and extraction solutions on cell quantifications in freshwater sediment, by comparing the results to those obtained with NaCl-free (0%) solutions. Cells were fixed according to the protocol of Langerhuus et al. (2012). Briefly, after retrieving the cores, fresh 0.5-cm^3^ sediment aliquots were taken using 3 mL cut-off syringes, homogenized with 0.5 mL of cell fixation solution (4% PFA, 3 or 0% NaCl), and incubated for 2–6 h at 4°C. PFA was then removed by washing twice with 1 mL PBS (3 or 0% NaCl) followed by centrifugation for 10 min at 10,000 × *g* and removal of supernatants. The final sediment pellet was resuspended in a 1:1 (*v/v*) PBS:ethanol solution, and stored at −20°C.

The cell extraction protocol is based on the protocol published by Langerhuus et al. (2012) and combines HF treatment, shaking and ultrasonication to destroy particles and release cells into suspension (Morono et al., 2013). Since little is known about how HF treatment might affect cell recovery and FCM counts on freshwater cell extracts, we evaluate the efficiency of HF treatment on lake sediments by (1) determining cell recovery rates based on sediment spikes with known numbers of *E. coli* cells, and (2) comparing the cell extraction efficiency of HF-based and density-gradient centrifugation-based assays (Histodenz) using natural samples. For the HF-based extraction protocol, we diluted the fixed sediment slurries, (e.g., 1:5) with 3% (or 0%) NaCl solution, and mixed 100 μL of this diluted sediment slurry with 600 μL of NaCl solution, 100 μL of detergent mix [100 mM ethylenediaminetetraacetic acid (EDTA), 100 mM sodium pyrophosphate, 1% (*v/v*) Tween 80, 3 or 0% NaCl], and 100 μL of methanol [Note: the composition of this mixture was based on Kallmeyer et al. (2008)]. Samples were then shaken for 60 min at 1,600 rpm using a ThermoMixer (Eppendorf, Hamburg, Germany). After shaking, the sediment slurries were sonicated at low intensity (∼160 W) for 20 min (10 cycles, 30 s on, and 30 s off) in an ice-water bath (Bioruptor^®^Plus, UCD-300, Diagenode, NJ, United States). 200 μL of 5% (*w/v*) HF (Sigma-Aldrich) was then added, and the samples were manually homogenized by shaking, and incubated at room temperature for exactly 20 min, with a second manual shaking after 10 min. The Histodenz-based extraction, which was followed by a final extraction step on the residual sediment pellet is explained later in this section (Other procedures, *Density-gradient centrifugation using Histodenz*).

#### Direct Staining

Direct staining of microbial DNA with SYBR-I is a standard and widespread procedure used for direct counting of microbial cells, and to distinguish microbial cells from particles, but results are not always reproducible by other methods, in particular when cell extracts are from sediments. We examined the relationship between cell quantification accuracy during direct staining and dye concentration, sample dilution, and sediment characteristics (TOC, grain size, marine vs. freshwater). Our tests include (1) applying the same staining condition (1 × SYBR-I, 1,000× sample dilution) on diverse sample types, and comparing the results to cell counts based on membrane-staining and subsequent FCM- and EFM-based quantifications (described in next sections); (2) using different dye concentrations (0.5×, 1×, 2×, 5×, 10×) on samples but keeping the sample dilution factor (1,000×) unchanged; (3) testing the effect of sample dilution (100×, 200×, 400×, 1,000×, 2,000×, 3,000×, 4,000×, 8,000×, 10,000×) on staining efficiency at constant dye concentration, (e.g., 1 × SYBR-I).

Tests were done by, immediately after the 20-min HF treatment, mixing 10–250 μL of cell extracts (will result in different sample dilution) at a ratio of 1:1 with STOP solution (1 M Tris–HCl, pH 8.0; 0.125 M CaCl_2_ and 25% methanol). Tris-EDTA (TE) buffer (10 mM Tris–HCl, pH 7.5; 1 mM EDTA, pH 8.0), and SYBR-I (provided as 10,000× in anhydrous dimethylsulfoxide, Sigma-Aldrich, St. Louis, United States) were then added to a final volume of 1 mL, and a final dye concentration of 0.5–10 × SYBR-I. Cells were stained in the dark for 15 min. Prior to FCM analysis, the mixture was sieved through a 35-μm nylon mesh (Corning, NY, United States) to remove remaining large particles. Multifluorescent microspheres (0.5 μm, excitation/emission maxima of 377/479, 517/546, 588/612 nm, Polysciences, Inc., PA, United States) were added for volumetric calibration at a concentration of 1.8–3.6 × 10^5^ beads mL^−1^.

#### Membrane Staining

For membrane staining, cell extracts were treated in the same way as for direct staining, except that the staining procedure took place after the cell extracts had been filtered onto a membrane. Cells were collected onto the membrane by following the filtration protocol of Weinbauer et al. (1998): a filtration tower was assembled by successively placing the cellulose acetate membrane (0.45-μm pore, 25-mm Ø, Whatman, United Kingdom), polycarbonate membrane (0.22-μm pore, 25-mm Ø, Whatman, United Kingdom), and filtration funnel onto the filter holder (Sterlitech corporation, Kent, United States). Depending on the cell numbers of the sample, 10–100 μL of cell extracts were diluted in 3 mL of TE buffer and pipetted to the filtration funnel. A vacuum pump (KNF LABOPORT^®^, Trenton, United States) was connected to the filtration tower to accelerate the filtration process. After collecting the cells onto the polycarbonate membranes, the membranes were stained in the dark for 15 min with 100 μL of 250 × SYBR-I. Afterward membranes were destained to remove excess SYBR-I solution by washing with TE buffer. This was done by distributing 1,000 μL of TE buffer across the entire membrane and subsequently removing this TE buffer, which now contained excess SYBR-I, by filtration. Next membrane was cut into 2 equal pieces, of which one piece was stored at −20°C for later EFM enumeration. The other half was then submerged in 5 mL of TE buffer within a 15 mL centrifuge tube. Cells were detached from the membrane into TE buffer using the Bioruptor sonicator at 160 W for 2 min (two cycles of 30 s on and 30 s off). The solution was then sieved through a 35-μm nylon mesh, diluted with TE buffer if necessary, and mixed with calibration beads as described above.

#### FCM Settings

We examined effects of FCM flow speed (10, 30, 60 μL min^−1^) and acquisition time (T = 1, 3, 5 min) on cell counts. In addition, we quantified potential effects of “apparatus coincidences,” i.e., particles arriving at the detection point of FCM coincidently with cells (Keij et al., 1991), by varying the rates of events from 20 to >10,000 events per second. Samples were analyzed using a Gallios flow cytometry system (Beckman Coulter, Brea, CA, United States) with multi-lasers (emitting light at 405-, 488-, 561-, 635-nm), and multiple detectors, i.e., green fluorescence was detected in the FL1 channel (525/30 BP), red fluorescence in the FL4 channel (695/30 BP), orange fluorescence in the FL3 channel (630/30 BP), forward scatter light (FS), and side scatter light (SS). The following FCM settings were kept constant throughout all measurements: (1) the voltage for all channels was set to 500 V; (2) the set gain was 1 for the fluorescent channels of FL1-10, and 5 and 10 for the FS and SS channels, respectively; (3) the target particle size was <1 μm; and (4) FL1 was set to be the channel for discrimination (threshold = 1). For different detectors and sample types, minor adjustments of the above voltage and gain settings might be necessary to place microbial populations in appropriate positions on FCM cytograms. Data were processed with the Kaluza analysis software (Beckman Coulter). Logarithmic dot plots of FL1/FL4 (or FL1/FS) and FL2/FL3 were used to distinguish signals of stained cells and fluorescent beads from background noise of non-biological particles, respectively. Positive controls (*E. coli* cells) and negative controls (autoclave-sterilized sediment without visible cells) were used to determine the gate positions on FCM cytograms. Positive, i.e., cell-specific fluorescent signals are higher in green and lower in red intensity of fluorescence, whereas background fluorescence, e.g., from sediment particles, are lower in green fluorescence.

### Other Procedures

#### EFM Counting

After the same staining and destaining procedures as for membrane staining described before, membrane pieces were transferred onto a glass slide. 15 μL of anti-fading solution (50% PBS (0 or 3% NaCl), 49.9% glycerol, 0.1% p- phenylenediamine) was used as a mounting medium. For each filter, 10–20 fields spanning the entire slide were selected randomly and photographed using an epifluorescence microscope system (DM6000B, Leica, Wetzlar, Germany). Blue-light excitation (band-pass filter: BP480/40) and green-light emission (band-pass filter: BP527/30) were used. Images were imported to ImageJ (Schneider et al., 2012), converted to inverted gray images, and the thresholds were adjusted between 210 and 240 to eliminate interfering signals from sediment particles (less green and less bright). Afterward images were smoothed, watershed, and cells were counted automatically using the “Analyze Particles” function. The ImageJ-based counts showed good agreement with conventional eye counts.

#### Density-Gradient Centrifugation Using Histodenz

Cells were detached from the sediment particles based on the published protocol of Kallmeyer et al. (2008), and separated from sediment particles using a density-gradient centrifugation method (Histodenz, Frossard et al., 2016). 100 μL of diluted sediment slurry, (e.g., 1:5 with 3% NaCl solution) were mixed with 300 μL of 3% NaCl solution, and 50 μL each of both Detergent Mix and methanol. Mixtures were then shaken for 60 min at 1,600 rpm using the ThermoMixer, followed by ultrasonication at low intensity (∼160 W) for 20 min in an ice-water bath of the Bioruptor sonicator. After ultrasonication, cell suspensions were homogenized by brief vortexing, 500 μL Histodenz (50% *w/v*, 1.4 g mL^−1^, Sigma-Aldrich, St. Louis, United States) was carefully injected to the bottom of the tube using a 2.5-mL syringe with needle. The tube was centrifuged at 3,000 × *g* for 15 min, after which the supernatant and entire interface, including the uppermost part of the Histodenz layer, were transferred to a new tube. An aliquot (volume depended on the cell number in the sample) of the supernatant was filtered, stained, and counted by EFM as described before. The remaining sediment pellet was resuspended in 400 μL of 3% NaCl and 50 μL of each, Detergent Mix and methanol. After repeating the shaking and ultrasonication procedures, an aliquot of the cell suspension was also filtered, stained, and counted by EFM.

#### Quantification of Bacterial and Archaeal 16S rRNA Genes

##### DNA Extraction

All sediment DNA was extracted according to the modular method published by Lever et al. (2015). All samples except those from eutrophic lakes were extracted using lysis protocol II with the following specifications: 0.2 g sediment were placed into screw-cap microcentrifuge tubes filled to ∼15% (*v/v*) with 0.1 mm zirconium-silica beads and mixed with 100 μL of 10 mM sodium hexametaphosphate solution. Next, 500 μL of lysis solution I was added. Lake Lucerne samples were vortexed for 30 s horizontally at maximum speed on a Vortex Genie 2 (Scientific Industries, New York, United States), while Guaymas Basin sediment samples were homogenized for 30 s at 30 shakings per second on a Tissue Lyzer LT (Qiagen). Afterward samples underwent a chemical lysis incubation for 1 h at 50°C and 600rpm on a ThermoMixer (Eppendorf), washed twice with chloroform-isoamly alcohol (24:1), precipitated with ethanol-sodium chloride solution containing linear polyacrylamide (LPA) as a co-precipitant (20 μg LPA mL^−1^ extract), and purified using the Norgen Kit (Promega, Madison, WI, United States) according to the manufacturer instructions (for details, see Lever et al., 2015).

Because large amounts of co-extracted humic substances from sediments of the eutrophic Lake Baldegg and Lake Greifen enhanced DNA losses during silica column purification, sediment DNA from these two lakes was extracted according to lysis protocol III from Lever et al. (2015). This protocol includes a step to remove undesired humic substances, e.g., polyphenols, polysaccharides. The only differences to the protocol applied to Lake Lucerne were that after the first chemical lysis incubation, 500 μL of lysis solution II [2.5 NaCl, 2% (*w/v*) cetyl trimethylammonium bromide (CTAB), 0.1% (*w/v*) polyvinylpolypyrrolidone (PVPP)] were added per sample, and the new mixture was incubated for an additional hour on the ThermoMixer at the same setting (50°C, 600rpm), after which a third 1-h incubation (now at 65°C, 600rpm) was included to enhance removal of undesired compounds. Furthermore, due to the already high sodium chloride concentrations from lysis solution II, no additional sodium chloride was added during ethanol-LPA precipitation. More details on the modular extraction method and its lysis protocols can be found in Lever et al. (2015)

##### qPCR

Concentrations of bacterial and archaeal 16S rRNA genes in DNA extracts were quantified on a Roche Light Cycler 480 II (Roche Molecular Systems, Inc.) by SYBR-Green based qPCR as described in Lever et al. (2015). The primer pairs for Bacteria and Archaea were Bac908F_mod (5′- AACTCAAAKGAATTGACGGG-3′) (Lever et al., 2015, modified from Ohkuma and Kudo, 1998) / Bac1075R (5′- CACGAGCTGACGACARCC-3′) (Ohkuma and Kudo, 1998), and Arch915F_mod (5′-AATTGGCGGGGGAGCAC-3′) (Cadillo-Quiroz et al., 2006) / Arch1059R (5′-GCCATGCACCWCCTCT-3′) (Yu et al., 2005), respectively. qPCR reactions (10 μL) were composed of 5 μL of 2 × SYBR Green I Master (Roche), 1 μL of 1 μg μL^−1^ bovine serum albumin, 0.5 μL of 10 μM of each primer, 1 μL of molecular-grade water, and 2 μL of undiluted DNA extract. Plasmids of 16S rRNA genes from *Thermoplasma acidophilum* and *Holophaga foetida* were applied as archaeal and bacterial standards, respectively. The thermal cycler protocol consisted of: (1) enzyme activation and initial denaturation at 95°C for 5 min; (2) 35 cycles (Bacteria) and 40 cycles (Archaea) of (a) denaturation at 95°C for 10 s, (b) annealing at 60°C (Bacteria) and 55°C (Archaea) for 30 s, (c) elongation at 72°C for 15 s, and (d) fluorescence measurement at 72°C (Bacteria) and 81°C (Archaea) for 15 s; and (3) a stepwise melting curve from 95 to 55°C in 1 min to check for primer specificity. All standards and samples were measured in duplicate.

#### Grain Size and TOC Analysis

Grain size distributions were measured using a LS 13320 Multi-Wavelength Laser Diffraction Particle Size Analyzer with Polarization Intensity Differential Scattering (PIDS) technology (Beckman Coulter, Indianapolis, United States). ∼0.5 cm^3^ of surface sediment samples were dispersed in 3 mL of sodium monophosphate (NaPO_4_) prior to analysis and disaggregated by brief ultra-sonication. Each sample was measured for 90 s. For TOC analyses, 5–10 g of sediment were freeze-dried, homogenized, and decarbonized with 3N hydrochloric acid for 24 h, and then dried and homogenized again for TOC analysis with a 1112 Flash Elemental Analyzer connected to a Delta V isotope ratio mass spectrometer (both Thermo Fisher Scientific, Bremen, Germany). Samples were wrapped in tin capsules and combusted at 1,030°C in an oxygen atmosphere. The system was calibrated using a standard of Atropine containing 70.56% (weight %) carbon.

## Results

### Impact of Salinity in Fixation and Extraction Solutions

**FIGURE 1:**
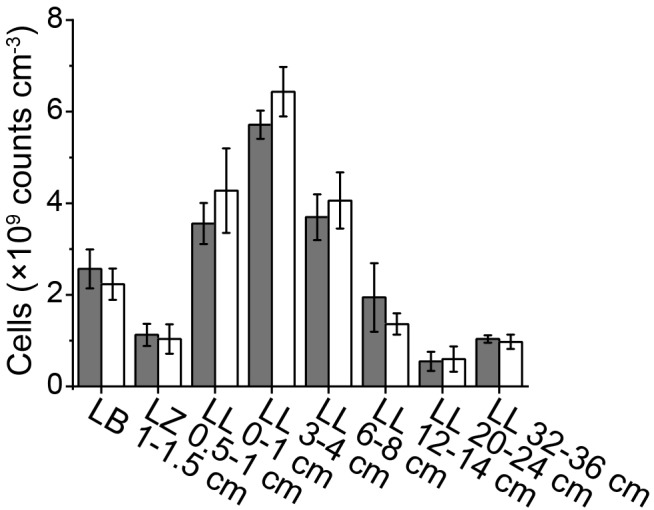
Cell abundances determined using PFA, PBS, detergent mix with (gray bars) or without 3% NaCl (white bars). Representative samples were taken from Lake Baldegg (LB), Lake Zurich (LZ), and Lake Lucerne (LL). Error bars represent the standard deviations of triplicates.

The presence of up to 3% NaCl in various extraction solutions had no significant effect on cell extraction efficiency from lake sediments (Figure 1; *p* > 0.05, *n* = 24, pairwise *t* test). Therefore, we from then on used fixation and extraction solutions with 3% NaCl for marine and freshwater sediment.

### Application of HF Treatment to Lake Sediment Samples

**FIGURE 2:**
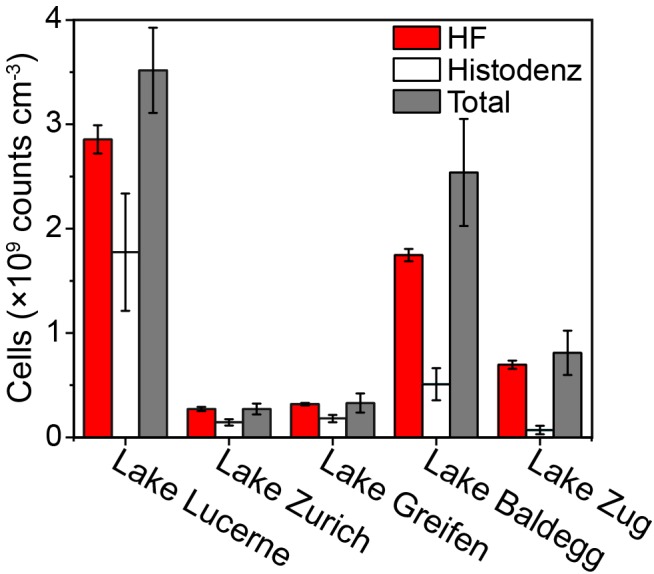
Comparison of cell counts using HF- and Histodenz-based assay for cell extraction. The total cell abundance represents the sum of cells extracted by Histodenz plus those remaining in the sediment pellet after extraction. Surface sediments (0.5–1 cm) from different lakes were tested, the standard deviations are derived from triplicates.

While HF treatment has long been used to extract cells from soils (Boenigk, 2004) and marine sediments (Morono et al., 2009; Langerhuus et al., 2012), it has to our knowledge not been applied to lake sediments. Microscopic examinations found that after HF treatment microbial cells from both autoclave-sterilized sediments spiked with *E. coli* cultures and natural lake sediments maintained their integrity and showed bright fluorescence (Supplementary Figure S1). In addition, cell recovery rates determined by spiking sterilized sediments with a known-number of *E. coli* cells, were 94.5 ± 12.2% (*n* = 6) after HF treatment, showing that there is no significant cell loss due to the harsh HF treatment. Compared to the Histodenz-based extraction protocol, the HF-based protocol consistently generated higher cell counts with lower standard deviations (Figure 2). If we assume that the sum of cells counted in supernatants plus sediment pellets after density-gradient centrifugation accurately reflect the actual cells numbers in our samples, the estimated extraction efficiency of HF-based protocol are 86.7 ± 12.7% (*n* = 5, each sample analyzed in triplicates) of all the tested samples. This is significantly higher than for Histodenz-based extraction, where the estimated extraction efficiency was only 37.4 ± 21.5% (*n* = 5, each sample analyzed in triplicates).

### Direct Staining Requires Optimization

**FIGURE 3:**
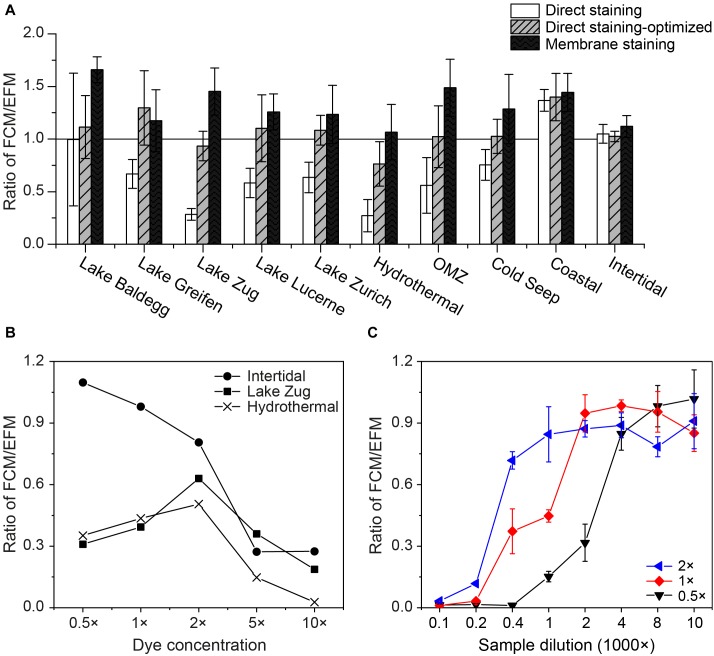
**(A)** Comparisons of FCM counts using different staining approaches: direct staining of using 1 × SYBR-I, 1000× sample dilution (white bars); direct staining after optimization, i.e., using D/S ratios of 2,000–8,000 (gray bars with slashes); membrane staining (black bars). To facilitate the comparisons, all of the FCM counts were normalized to the corresponding EFM counts by dividing FCM- by EFM-based numbers. Hence, a value of 1.0 indicates equal cell counts by FCM and EFM. At least three samples from each location shown in Table 1 were selected for tests. **(B)** Relationship between the dye concentrations used for direct staining and their FCM counts. Samples from Lake Zug, Hydrothermal area, and intertidal zone were selected, due to the extremely low (or high) staining efficiencies of these samples found in the initial tests. **(C)** Impact of sample dilution on direct staining of cell extracts, when 0.5×, 1×, 2 × SYBR-I were applied. Sediments from Lake Zug (6–8 cm) were used for tests. Error bars are standard deviations derived from triplicates.

Applying the same dye concentration and sample dilution (1 × SYBR-I, 1000× sample dilution) during direct staining resulted in FCM counts that were inconsistent with EFM counts across the ten locations tested (FCM/EFM = 0.3–1.4, Figure 3A). FCM counts on directly stained cell extracts from the more coarse coastal and intertidal sediment samples (N Gulf of California, False Bay; Table 1) agreed well with EFM counts. In all other cases FCM counts after direct staining were significantly lower than EFM counts (*p* < 0.05, *n* = 24, pairwise *t* test), with the greatest discrepancies occurring in the samples from Lake Zug and hydrothermally altered sediment (Guaymas Basin).

We thus tested different dye concentrations in an attempt to improve the staining efficiency of these samples (Figure 3B). Interestingly, when we increased the dye concentrations from 0.5× to 2 × SYBR-I, the FCM counts of Lake Zug and hydrothermal sediment increased, followed by clear drops when SYBR-I concentrations were raised further to 5× and 10×. In contrast, the FCM counts on intertidal sediment decreased at higher dye concentrations, indicating that potentially the more coarse sample lithology of intertidal sediment compared to fine lake or hydrothermally altered sediment affected the optimum dye concentration. The sample-specific differences in the impact of dye concentrations on cell counts can also be seen in the cytograms of intertidal and Lake Zug sediment (Supplementary Figure S2).

Further tests showed, that, in addition to dye concentration, sample dilution exerts a clear impact on FCM counts: as the dilution factor of Lake Zug cell extracts increased from 100 to 10,000×, the FCM counts increased (Figure 3C). Yet, this effect apparently co-varied with dye concentrations, i.e., using higher dye concentration, (e.g., 2 × SYBR-I) satisfactory cell counts can be obtained at lower sample dilution, (e.g., 1,000× sample dilution). Given the fact that FCM counts based on direct staining were significantly co-influenced by dye concentration, sample dilution, and sample lithology, we therefore performed sample type-specific optimizations on the dye concentration and/or sample dilution. The used optimal staining conditions varied with sample types and were shown as the D/S ratios, i.e., ratio of dye concentration to sediment amount in Table 1 (for example, 1 × SYBR-I and 0.25 × 10^−3^cm^−3^ sediments result in D/S = 4,000, see Supplementary Table S1 for more information). After the optimizations, the direct-staining based FCM counts on different types of samples were significantly improved and thus show excellent agreement with the EFM counts (average ratio: 1.08 ± 0.27; pairwise *t-test p* > 0.05, *n* = 31).

We further explored potential relationships between dye concentration, sample dilution, and sediment characteristics by plotting optimal D/S ratios of the ten sample types shown in Table 1 against sample-specific clay+silt and TOC contents (Supplementary Figure S3). D/S ratios showed a significant, positive correlation with clay+silt contents (*r^2^* = 0.55, *p* < 0.05, *n* = 10, liner regression), i.e., more dye and/or less sediment amount were required to reach the optimal cell counts when samples contain higher percentages of clay+silt (Supplementary Figure S3A). Yet such relationship between optimal D/S ratios and TOC contents is not statistically significant (Supplementary Figure S3B).

### Membrane Staining Provides Bright Staining

**FIGURE 4:**
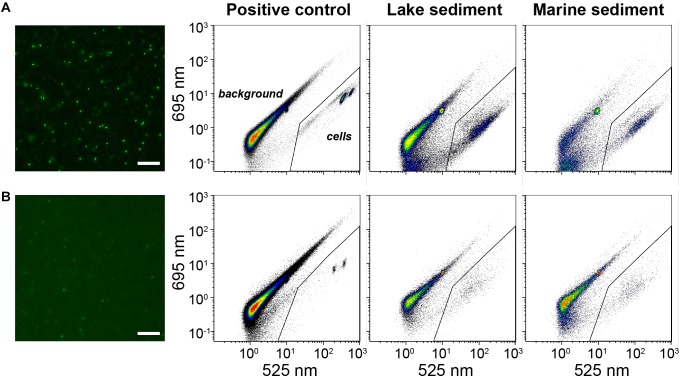
Comparisons of microscopic images and flow cytometric cytograms based on **(A)** membrane staining and **(B)** direct staining. Representative samples include positive control (autoclave-sterilized sediments spiked with *E. coli* cells), lake, and marine sediments. The gate (solid line) was set to discriminate the signals of stained cells from background signals. White bar: 25 μm.

As an alternative to direct staining of cell extracts, we filtered and stained the cells on polycarbonate membrane, to enhance the cell staining. Since EFM and membrane-based FCM counting share exactly the same procedures for cell extraction, filtration, and staining, to save time and cost, one piece of black polycarbonate membrane was employed for cell filtration and staining, and afterward was cut into two equal pieces, of which one was used for EFM and the other for FCM analyses. By performing 2-min sonication, the stained cells can easily be detached from the polycarbonate membrane back to TE buffer for further FCM analysis (Supplementary Figure S4), and the calculated cell recovery rates in this process are high (93.4 ± 5.4%, *n* = 6). As expected, FCM counts based on membrane staining overall agree with the corresponding EFM counts, which share the same staining procedure (Figure 3A). Average FCM counts after membrane staining are even slightly higher (average ratio: 1.32 ± 0.27; pairwise *t-test p* < 0.05, *n* = 31) than ones after EFM counts, possibly due to the higher sensitivity of the FCM method. Similarly, FCM counts based on membrane staining are in good agreement with FCM counts after sample-optimized direct staining (average ratio: 1.28 ± 0.36; pairwise *t-test p* < 0.05, *n* = 31). Further comparisons between the techniques of membrane and direct staining show that membrane staining provides stronger staining, and thus fluorescent signals, of microbial cells, which facilitates the gate setting and discriminations between stained cells and background signals on cytograms (Figure 4).

### FCM Settings

Although our tests on FCM settings are instrument-specific, they might provide procedural insights to other users. For data acquisition, we did not find significant differences among cell number estimates produced at flow speeds of 10, 30, and 60 μL min^−1^ (3.61 ± 0.07 × 10^9^, 3.66 ± 0.12 × 10^9^, 3.64 ± 0.16 × 10^9^ cells cm^−3^, respectively; *p* > 0.05, Kruskal-Wallis test), even though we recommend low flow speeds because of the slightly lower standard deviations. At low flow speed (10 μL min^−1^), the acquisition time (T = 1, 3, 5 min) did not affect the cell counts significantly (2.05 ± 0.07 × 10^9^, 1.99 ± 0.07 × 10^9^, 2.04 ± 0.02 × 10^9^ cells cm^−3^, respectively; *p* > 0.05, Kruskal-Wallis test). Overall, there was no significant difference in cell counts when rates of events varied between 20 to >10,000 events s^−1^ (Supplementary Figure S5; *p* > 0.05, Kruskal-Wallis test). The well-known “apparatus coincidences” effect thus appeared to exert only a minor impact on cell enumerations using the Gallios FCM system. Yet, extremely high (>10,000 events s^−1^) or low (<50 events s^−1^) speeds of events generated larger standard deviations, whereas optimal counts, with the lowest standard deviations, were obtained at a speed of ∼900 events s^−1^. Different gating strategies, i.e., plotting green fluorescence (525/30 nm) against red fluorescence (695/30 nm) or against forward scatter light (FS) produced highly consistent results (Supplementary Figure S6).

### Comparison of FCM and EFM Counts Across Additional Samples

**FIGURE 5:**
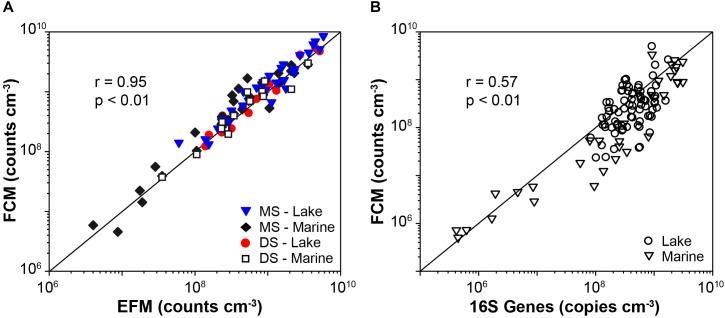
**(A)** Comparisons of cell abundances determined by EFM and FCM enumerations, which involved results based on both membrane staining (MS) and direct staining (DS), in lake and marine samples (46 samples). Solid lines indicate 1:1 lines. **(B)** Compare 16S gene rRNA abundances determined by qPCR to FCM counts of lake and marine sediment based on the membrane staining technique (*n* = 110).

FCM counts based on both membrane staining and direct staining after sample-specific optimizations show good agreements with EFM counts, across samples from diverse habitats (20 different locations in total) that differ greatly in microbial population size (*r* = 0.95, *p* < 0.01, *n* = 92, Pearson correlation; Figure 5A and Supplementary Table S2). Notably, for each sample type (location), only one sample was used for testing the optimal conditions of dye concentration and sample dilution. This optimized dye concentration and dilution was then used for all other samples from the same location (for information of all samples, see Supplementary Table S2).

### FCM vs. qPCR

We successfully applied the optimized FCM enumeration protocol on exploring the distribution of microbial abundances in both lake and marine sediment, and the FCM data show significant relation to the qPCR data (Figure 5B; *r* = 0.57, *p* < 0.01, *n* = 110, linear regression). The calculated 16S genes per cell are 2.4 ± 2.6 for lake samples (*n* = 76), and 3.5 ± 3.8 for marine samples (*n* = 34). These calculations indicate excellent agreement between the FCM and qPCR data, given the global mean ± standard deviations of 16S genes per cell is 4.7 ± 2.8 for Bacteria and 1.7 ± 0.9 for Archaea^1^.

## Discussion

**FIGURE 6:**
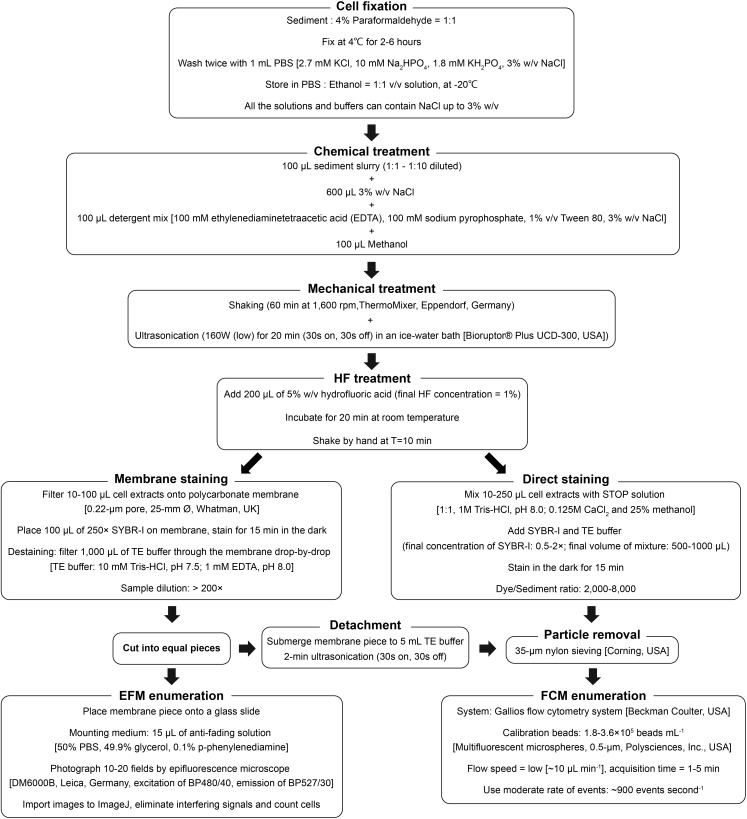
Schematic diagram of the final protocol used for FCM and EFM enumeration of microbial cells in sediment samples.

Based on various tests and optimizations in this study, a final protocol including both direct and membrane staining procedures for the quantification of microbial populations in sediment is proposed (Figure 6). This protocol is applicable to a wide range of marine and freshwater sediments and produces good agreement between (1) high-throughput (direct) and low-throughput (membrane) staining protocols (Figure 3A, 5A), (2) FCM- and EFM-based counts (Figure 3A, 5A), and with (3) an independent, DNA-based microbial quantification method (Figure 5B). By testing and comparing the results of direct staining and membrane staining we demonstrate the crucial but widely overlooked importance of sample-specific optimization during the use of direct staining protocols.

To date, various techniques for cell detachment and separation have been implemented. For instance, density-gradient centrifugation effectively separates cells from sediment particles and is hence suitable for extracting and concentrating cells from low-biomass samples; yet, a high cell extraction efficiency is difficult to attain, and requires excellent handling skills (Amalfitano and Fazi, 2008; Kallmeyer et al., 2008; Frossard et al., 2016). HF treatment has been reported to significantly enhance cell extraction efficiency and accelerate the cell extraction process (Boenigk, 2004; Morono et al., 2009; Langerhuus et al., 2012). Our study confirms this observation and shows that HF treatment is suitable across a wide range of lithologically distinct marine and lake sediment types that differ by over three orders of magnitude in microbial population size (Table 1 and Figure 5). We, furthermore, show that HF treatment can be combined with direct staining protocols in flow cytometric applications, which enables a much higher sample throughput (60–80 samples per 8-h day) than the more time- and labor-intensive membrane staining approach with FCM (20–30 samples), or EFM (8–10 samples).

While considerable efforts have been invested in improving the cell extraction efficiency from environmental samples (references in Introduction), our results indicate that the optimization of staining efficiency of the extracted cells is an additional important parameter that has frequently been neglected. The widely used, time-efficient technique of direct staining is in principle ideal to couple with FCM enumeration, however, it is prone to inaccuracies as a result of suboptimal dye concentrations and sample dilutions both of which depend on lithology (Figure 3). We show that there is no universally ideal dye concentration or sample dilution, and that when the same dye concentration and sample dilution is applied to different sample types, e.g., coarse-grained, low-TOC vs. fine-grained, organic-rich sediment, the cell staining efficiency may vary significantly. Though more tests are necessary for additional verification, our data suggest that optimum dye/sediment ratios are higher for samples with a high (>85%) clay+silt content than for more coarse-grained samples with higher contents of sand (Table 1 and Supplementary Figure S3A).

By contrast, the more time-consuming and labor-intensive membrane staining method, which requires an extra ≥15 min of processing per sample and the purchase of membrane filters and filtration equipment, is more robust across sample dilutions and sample lithologies and does not require initial optimization steps (Figure 3A, 4). This might be because during membrane staining concentrated SYBR-I (250×) can be used to ensure good labeling of all cells, and subsequently excess dye can be eliminated by destaining. Furthermore, small fluorescent particles (<0.2 μm diameter) can pass through the 0.2-μm membrane filter. It is likely that more consistent labeling of cells and reduction of unspecific labeling of background particles produces slightly more reliable results with membrane staining, even when compared to optimized direct staining (Figure 4).

## Conclusion

We present a versatile, accurate, and detailed flow cytomeric protocol for the quantification of microbial populations in sediments by direct staining or membrane staining (Figure 6). Assuming necessary instrument setups are available, we recommend membrane staining for sample sets with highly diverse sample types and/or small sample sizes, where the initial time invested into optimizing the direct staining procedure is not rewarded by downstream time-savings. By comparison, we recommend direct staining for large batches of samples with similar sediment properties. Here, after the initial optimization, the same dye concentration and sample dilution can be efficiently applied across all samples, skipping the need for time- and labor-intensive filtration, and resulting in significant cost savings with respect to instrument and consumable purchases. Although the decision on which staining protocol to use largely depends on needs and purposes, our study shows that after optimization, both staining protocols produce high-quality cell counts which agree well with each other, with corresponding EFM counts, and with independent, PCR-based quantification methods.

## Author Contributions

ML and LD designed the research. LD performed the research. LD, AF, XH, ND, and SB analyzed the data. LD and ML wrote the manuscript. All co-authors reviewed and commented the manuscript.

## Conflict of Interest Statement

The authors declare that the research was conducted in the absence of any commercial or financial relationships that could be construed as a potential conflict of interest.

## Footnotes

1 https://rrndb.umms.med.umich.edu/
