# Supplementary material for: Improving the Accuracy of Flow Cytometric Quantification of Microbial Populations in Sediments: Importance of Cell Staining Procedures
Source: Front Microbiol. 2019 Apr 9;10:720. doi: 10.3389/fmicb.2019.00720 (PMC6465615; doi:10.3389/fmicb.2019.00720)
Supplement: Supplementary file 1 [file Data_Sheet_1.docx]

Supplementary Material

Improving the accuracy of flow cytometric quantification of microbial populations in sediments: importance of cell staining procedures

Longhui Deng^1^*, Annika Fiskal^1^, Xingguo Han^1^, Nathalie Dubois^2,3^, Stefano Bernasconi^3^, Mark Alexander Lever^1^*

*** Correspondence:**

Longhui Deng

[longhui.deng@usys.ethz.ch](mailto:longhui.deng@usys.ethz.ch)

Dr. Mark Alexander Lever

[mark.lever@usys.ethz.ch](mailto:mark.lever@usys.ethz.ch)

**Supplementary Figure S1.** Microscopic images of the positive control (sterile sediments spiked with E. coli) without (A-1) and with (A-2) HF treatment; and natural samples from Lake Baldegg without (B-1) and with (B-2) HF treatment. White bar: 25 μm.


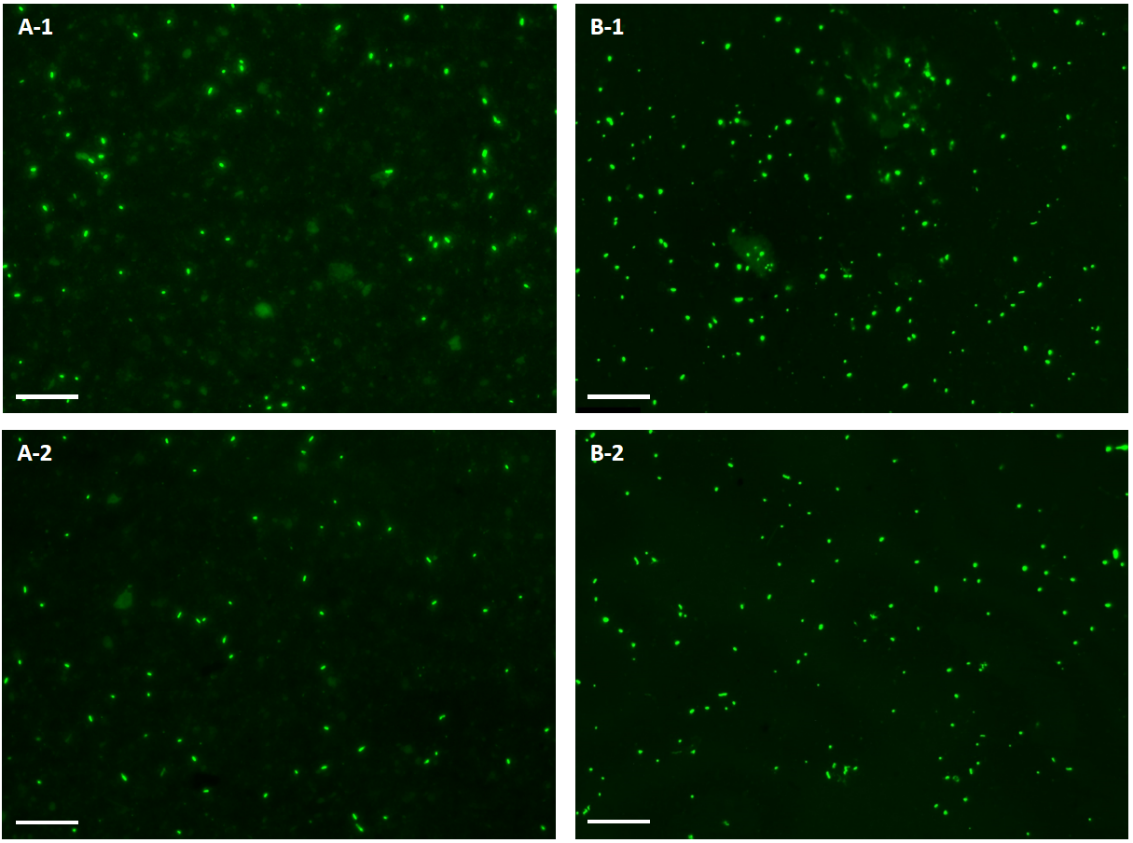


**Supplementary Figure S2.** Representative cytograms for testing the effect of dye concentrations on direct staining of: (A) Intertidal sediment; (B) Lake Zug sediment.
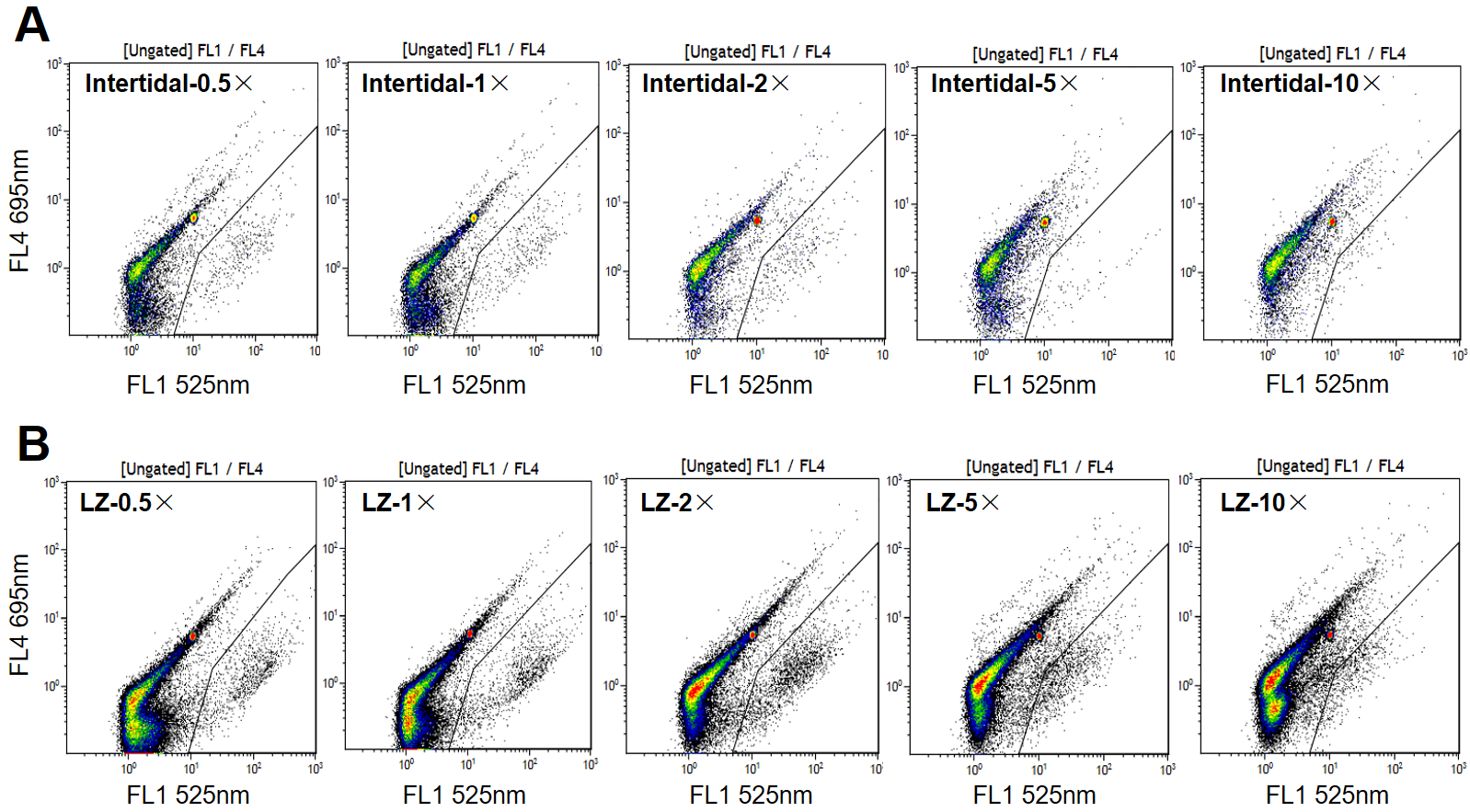


**Supplementary Figure S3.** Correlations between the optimal D/S ratios and (A) percentage of fine particles (clay + silt), (B) optimal D/S ratios and TOC, respectively. The optimal D/S ratios are the ratios of dye concentration to sediment volume we used, to reach the optimal cell counts. The solid lines represent the liner regression lines.


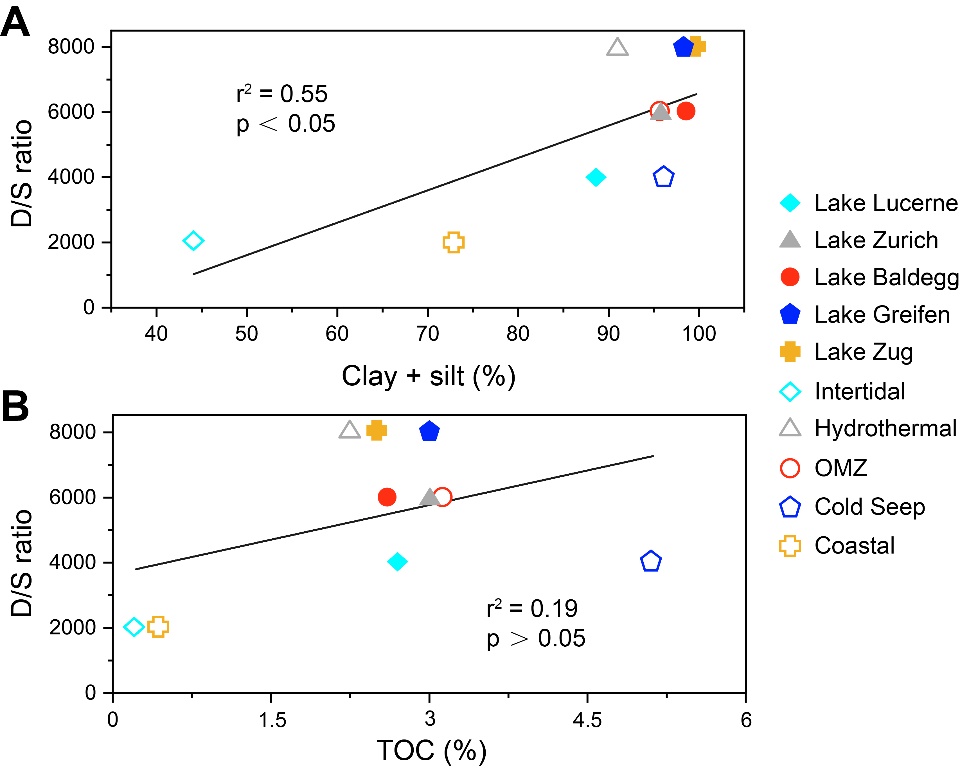


**Supplementary Figure S4.** Microscopic examinations of the polycarbonate membrane, before and after cells were detached from the membrane into TE buffer by 2-min ultrasonication.


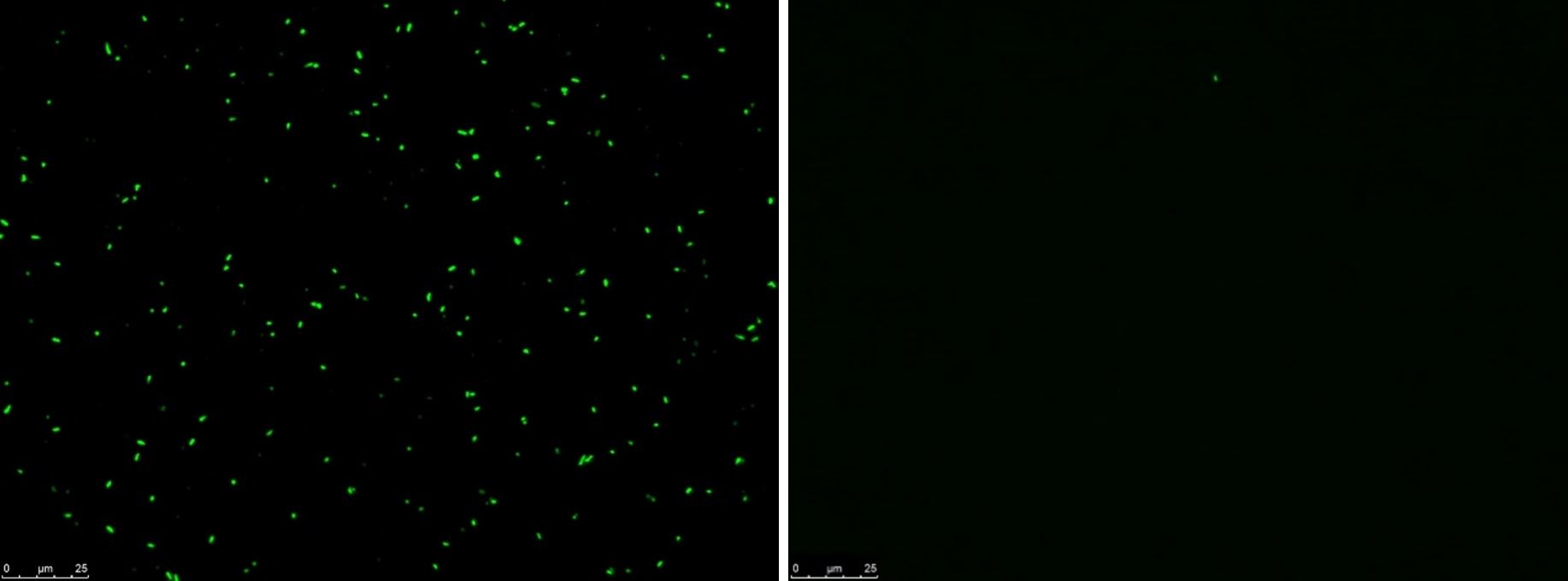


**Supplementary Figure S5.** Cell numbers measured in different rates of events. Sediment samples (2.5×10^-3^ cm^3^) from Lake Lucerne were stained on membrane with 250×SYBR-I, detached into TE buffer, and diluted serially. For event rates of 4,410 and 11,442, medium and high flow speed were used. Error bars represent the standard deviations of triplicate measurements.


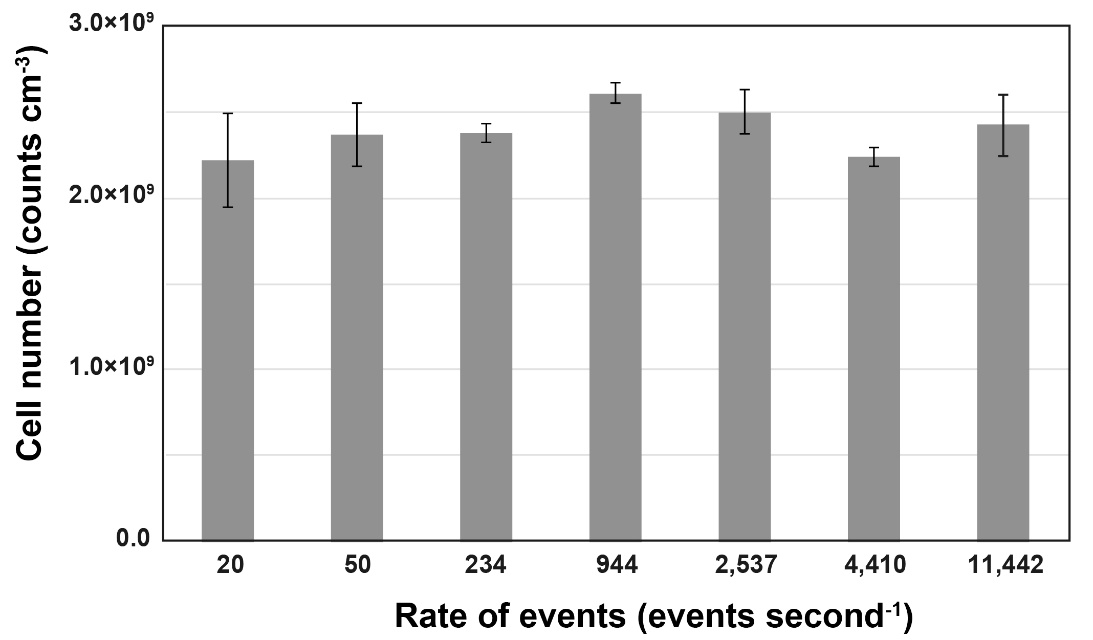


**Supplementary Figure S6.** Representative FCM cytograms of the positive control (sterilized sediments spiked with E. coli cells), lake and marine samples. The SYBR-stained cells were distinguished from the background fluorescence by plotting: (A) green fluorescence (525/30 nm) against red fluorescence (695/30 nm); (B) green fluorescence (525/30 nm) against forward scatter light (FS).


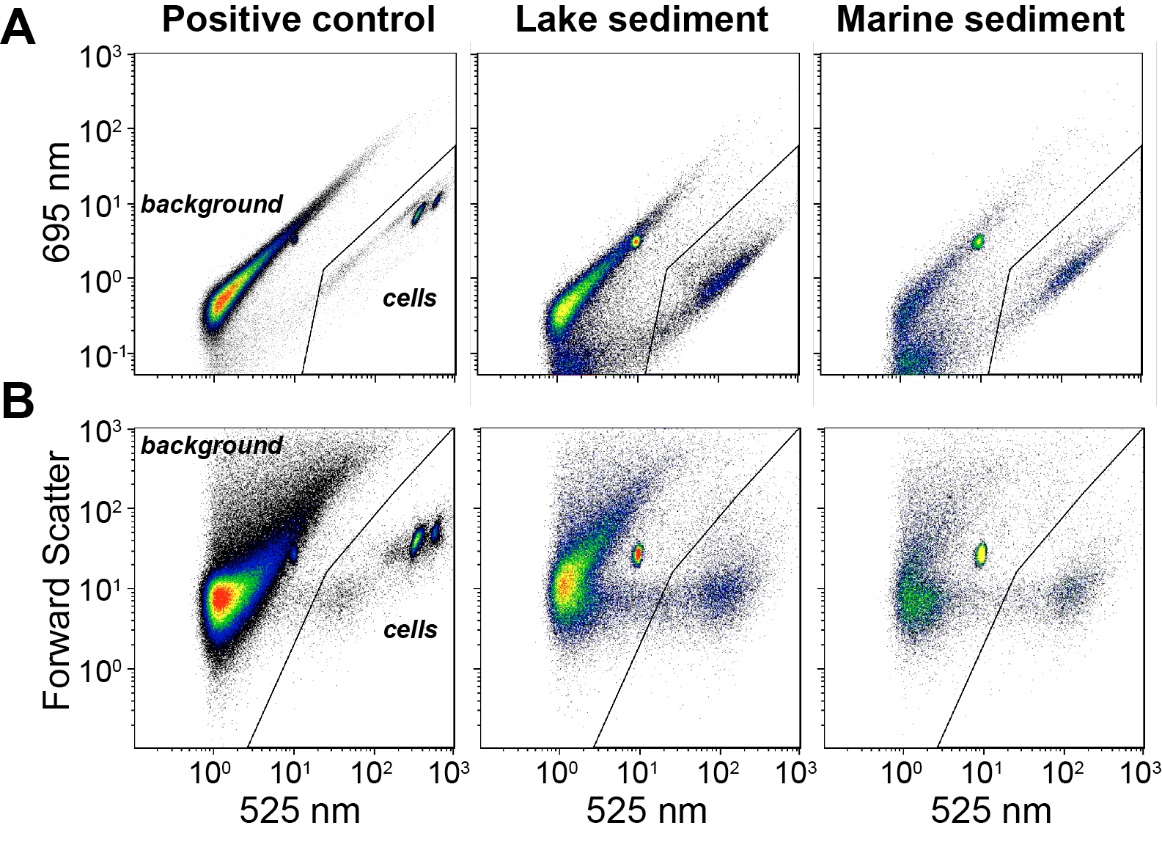


| **Samples** | **Sample depths (cm)** | **D/S ratio** | **SYBR-I** | **Sediment volume (cm^-3^)** |
| --- | --- | --- | --- | --- |
| **Lake Sediment** |  |  |  |  |
| Lake Lucerne (LL) | 1-1.5 | 4,000 | 1× | 0.250×10^-3^ |
| Lake Zurich (LZ) | 1.5-2 | 6,000 | 1× | 0.167×10^-3^ |
| Lake Greifen (LG) | 1-1.5 | 8,000 | 1× | 0.125×10^-3^ |
| Lake Zug (LZG) | 1-1.5 | 8,000 | 1× | 0.125×10^-3^ |
| Lake Baldegg (LB) | 1-1.5 | 6,000 | 1× | 0.167×10^-3^ |
| **Marine Sediment** |  |  |  |  |
| False Bay (intertidal) | 0-2 | 2,000 | 0.5× | 0.25×10^-3^ |
| Guaymas Basin (hydrothermal) | 0-1 | 8,000 | 1× | 0.125×10^-3^ |
| Guaymas Basin (OMZ) | 0-5 | 6,000 | 1× | 0.167×10^-3^ |
| Guaymas Basin (cold seep) | 33 | 4,000 | 1× | 0.250×10^-3^ |
| N Gulf of California (coastal) | 0-1 | 2,000 | 0.5× | 0.25×10^-3^ |

**Supplementary Table S1.** Samples used for sample type-specific optimization during direct staining. The optimal conditions for direct staining are shown as D/S ratios, i.e. the ratio between SYBR-I concentration and sediment volume used for staining.

**Supplementary Table S2.** Overview of sample types (locations), sample depths, and cell numbers counted by membrane-staining based method (FCM-M), direct staining after sample type-specific optimization (FCM-D), and by epifluorescence microscopy (EFM). Samples used for testing the direct staining protocol in Fig. 3 are marked by asterisks.

| **Locations** | **Latitude (N)** | **Longitude** | **Station** | **Depth (cm)** | **FCM-M** | **FCM-D** | **EFM** |
| --- | --- | --- | --- | --- | --- | --- | --- |
| Lake Baldegg (LB) | 47° 11.93' | 8° 15.61' E | 1 | 1-1.5* | 1.46E+09 | 1.13E+09 | 8.69E+08 |
|  |  |  | 1 | 1.5-2 | 1.14E+09 | - | 1.77E+09 |
|  |  |  | 2 | 1.5-2 | 2.88E+09 | - | 1.65E+09 |
|  |  |  | 1 | 4-6* | 1.86E+09 | 1.34E+09 | 1.05E+09 |
|  |  |  | 1 | 28-32* | 4.86E+08 | 2.44E+08 | 3.18E+08 |
| Lake Greifen (LG) | 47° 21.13' | 8° 40.51' E | 1 | 0-0.5 | 2.51E+09 | - | 1.54E+09 |
|  |  |  | 1 | 1-1.5* | 4.52E+09 | - | 3.66E+09 |
|  |  |  | 1 | 1.5-2 | 6.70E+08 | - | 1.13E+09 |
|  |  |  | 1 | 6-8* | 3.42E+08 | 4.00E+08 | 2.39E+08 |
|  |  |  | 1 | 28-32* | - | 1.91E+08 | 1.55E+08 |
| Lake Lucerne (LL) | 47° 00.05' | 8° 20.22' E | 1 | 1-1.5* | 1.43E+09 | 1.05E+09 | 1.31E+09 |
|  |  |  | 2 | 1-1.5* | 4.10E+09 | 4.14E+09 | 2.77E+09 |
|  |  |  | 1 | 1.5-2 | 2.40E+09 | - | 2.35E+09 |
|  |  |  | 1 | 4-6 | 1.01E+09 | - | 4.63E+08 |
|  |  |  | 1 | 6-8* | 5.90E+08 | 5.56E+08 | 4.55E+08 |
|  |  |  | 1 | 10-12 | 3.17E+08 | - | 2.95E+08 |
|  |  |  | 1 | 28-32* | 1.61E+08 | 1.23E+08 | 1.39E+08 |
|  |  |  | 2 | 28-32 | 1.41E+08 | - | 6.02E+07 |
|  |  |  | 3 | 28-32 | 2.24E+08 | - | 2.65E+08 |
| Lake Zug (LZG) | 47° 10.27' | 8° 30.04' E | 1 | 0-0.5 | 1.57E+09 | - | 1.68E+09 |
|  |  |  | 2 | 1-1.5* | 1.18E+09 | 7.64E+08 | 7.02E+08 |
|  |  |  | 3 | 0-0.5 | 2.14E+09 | - | 2.28E+09 |
|  |  |  | 1 | 4-6 | 2.45E+08 | - | 2.07E+08 |
|  |  |  | 2 | 6-8* | 7.09E+08 | 4.44E+08 | 5.46E+08 |
|  |  |  | 2 | 20-24* | 3.21E+08 | 2.10E+08 | 2.34E+08 |
| Lake Zurich (LZ) | 47° 17.00' | 8° 35.62' E | 1 | 0-0.5 | 6.19E+09 | - | 4.21E+09 |
|  |  |  | 1 | 1-1.5 | 8.52E+09 | - | 5.80E+09 |
|  |  |  | 1 | 1.5-2* | 6.94E+09 | 5.38E+09 | 4.51E+09 |
|  |  |  | 1 | 4-6* | 5.18E+09 | 4.76E+09 | 5.15E+09 |
|  |  |  | 2 | 28-32* | 1.08E+09 | 1.06E+09 | 9.37E+08 |
| Guaymas Basin (hydrothermal) | 27° 24.58' | 111° 23.27' W | 1 | 0-1* | 2.78E+09 | 1.10E+09 | 2.10E+09 |
|  |  |  | 1 | 14-16* | 2.83E+08 | 2.44E+08 | 2.63E+08 |
|  |  |  | 1 | 6-8* | 2.84E+09 | 2.99E+09 | 3.57E+09 |
| Guaymas Basin (OMZ^1^) | 27° 42.41' | 111° 13.66' W | 1 | 350* | 1.42E+09 | 1.01E+09 | 8.36E+08 |
|  |  |  | 1 | 0-5* | 3.82E+08 | 2.85E+08 | 2.42E+08 |
|  |  |  | 1 | 900* | 3.39E+08 | 1.96E+08 | 2.87E+08 |
| Guaymas Basin (cold seep) | 27° 28.19' | 111° 28.36' W | 1 | 483* | 1.03E+08 | 8.95E+07 | 1.06E+08 |
|  |  |  | 1 | 33* | 4.28E+08 | 3.97E+08 | 3.42E+08 |
|  |  |  | 1 | 233* | 3.80E+08 | 2.49E+08 | 2.33E+08 |
| N Gulf of California (coastal) | 27° 55.01' | 111° 01.13' W | 1 | 0-1* | 1.48E+09 | 1.50E+09 | 9.07E+08 |
|  |  |  | 1 | 4-6* | 2.99E+08 | 3.11E+08 | 2.36E+08 |
|  |  |  | 1 | 16-18* | 8.18E+08 | 6.96E+08 | 5.68E+08 |
| False Bay (intertidal) | 48° 29.20' | 123° 04.48' W | 1 | 6-8* | 2.85E+08 | 2.43E+08 | 2.31E+08 |
|  |  |  | 1 | 0-2* | 8.86E+08 | 8.37E+08 | 8.65E+08 |
|  |  |  | 1 | 26-30* | 3.92E+07 | 3.74E+07 | 3.55E+07 |
| Guaymas Basin (cold seep) | 27° 33.30' | 111° 32.88' W | 1 | 2-3 | 1.67E+09 | 9.87E+08 | 5.26E+08 |
|  |  |  | 1 | 1-2 | 2.05E+09 | - | 2.31E+09 |
| Guaymas Basin (reference) | 27° 26.52' | 111° 29.93' W | 1 | 1-2 | 5.31E+08 | - | 1.05E+09 |
|  |  |  | 1 | 4-6 | 2.04E+09 | - | 1.40E+09 |
| Guaymas Basin (graben) | 27° 23.83' | 111° 25.92' W | 1 | 1-2 | 1.41E+09 | - | 1.44E+09 |
| Guaymas Basin (cold seep) | 27° 28.16' | 111° 28.35' W | 1 | 1-2 | 2.22E+09 | - | 2.08E+09 |
| Guaymas Basin (below OMZ) | 27° 34.80' | 111° 21.54' W | 1 | 1-2 | 1.13E+09 | - | 3.98E+08 |
| Guaymas Basin (OMZ) | 27° 42.41' | 111° 13.66' W | 1 | 1-2 | 8.70E+08 | - | 3.24E+08 |
| Guaymas Basin (hydrothermal) | 27° 24.47' | 111° 23.38' W | 1 | 37 | 5.57E+07 | - | 2.87E+07 |
|  |  |  | 1 | 237 | 2.22E+07 | - | 1.76E+07 |
|  |  |  | 1 | 337 | 4.55E+06 | - | 8.73E+06 |
| Guaymas Basin (hydrothermal) | 27° 24.49' | 111° 23.38' W | 1 | 48 | 1.42E+07 | - | 1.89E+07 |
|  |  |  | 1 | 348 | 5.87E+06 | - | 4.03E+06 |
| Guaymas Basin (cold seep) | 27° 28.18' | 111° 28.40' W | 1 | 50 | 5.05E+08 | - | 4.47E+08 |
| Guaymas Basin (cold seep) | 27° 33.30' | 111° 32.88' W | 1 | 50 | 6.93E+08 | - | 3.40E+08 |
|  |  |  | 1 | 300 | 2.08E+08 | - | 1.01E+08 |
